# Akin Osteotomy: A Review of Modern Fixation Techniques

**DOI:** 10.7759/cureus.57026

**Published:** 2024-03-27

**Authors:** Amr H Eldessouky, Mohammad U Khattak, Ahmed M Srour

**Affiliations:** 1 Trauma and Orthopaedics, Worcestershire Acute Hospitals NHS Trust, Redditch, GBR

**Keywords:** osteotomy, akin, screw, suture, staple, hallux valgus

## Abstract

Akin osteotomy is commonly used to correct hallux valgus interphalangeus (HVI) deformity. The preferred implant for fixation remains an area of debate, often influenced by the surgeon's inclinations and expertise. This review compares the outcomes of contemporary fixation methods utilized in Akin osteotomy. PubMed served as the primary electronic database for the search. The interventions encompassed the use of screw(s), staple(s), and suture(s). The participants considered were adults aged 18 and above, undergoing Akin osteotomy either as a primary procedure or as an adjunct. Excluded were osteotomies performed via percutaneous or minimally invasive methods. Seven studies involving 590 feet were analysed, showcasing an impressive 99.8% overall union rate. The incidence of overall complications stood at 8.98%, with metal prominence notably higher in the screw fixation group (10.5%). All studies exhibited postoperative improvement in radiological angles. Screw, staple, and suture fixations demonstrated excellent union rates. While screws and staples offer robust osteotomy fixation, they pose risks of metal irritation and prominence. Suture fixation delivers comparable outcomes to the other two stabilization methods at a lower cost.

## Introduction and background

Hallux valgus is a common condition with a global incidence of 19% [1]. Patients present mainly with pain and discomfort, especially with footwear. Metatarsal osteotomies have long been established as the main surgical management for hallux valgus deformity [2]. Akin osteotomy is indicated explicitly for persistent interphalangeal angle (IPA) deformity following soft tissue balancing and osseous procedures to correct the intermetatarsal angle, distal metatarsal articular angle, and hallux valgus angle [3]. Kaufmann et al. found that an 8° proximal-distal phalangeal articular angle (PDPAA) reliably determines when an Akin osteotomy is recommended [4].

The initial description of the Akin osteotomy dates back to 1925, detailing the resection of the medial exostosis of the first metatarsal head and performing a closed wedge osteotomy at the base of the proximal phalanx [5]. Various alterations have been introduced since that time [6-8]. Most authors agree that relying solely on the Akin osteotomy for correcting hallux valgus deformity is not recommended [9,10].

Internal fixation has been the preferred approach to maintain the alignment for Akin osteotomy. Several techniques have been developed to achieve this goal, including intraosseous wire loops, crossed Kirschner (K) wires, sutures, screws, locking plates, and staples [11-16]. K-wire fixation is rarely used nowadays due to the risk of infection and the necessity for removal [3,9]. Screw fixation provides good compression, while staples are probably the less technically demanding procedure. Screws, staples, and wire loops all carry risk of metalwork irritation [17,18]. Multiple studies have demonstrated positive results using the suturing technique, which avoids soft tissue irritation caused by metalwork and can be performed using simple equipment. However, surgery using this method can be a bit fiddly, and there have been concerns about the stability of the fixation [13,19,20].

Akin osteotomy is a reliable surgery with union rates reported as high as 100% [16,18]. Complications are infrequent and appear to be correlated with the choice of fixation method. The available literature primarily consists of retrospective studies concentrating on a specific fixation method. We have come across only one report that compares the three most prevalent fixation methods [16], but there is no available systematic review on this topic. Accordingly, this review aims to consolidate and compare the modern fixation techniques used in Akin osteotomy.

## Review

Methods

Search Technique

**Figure 1:**
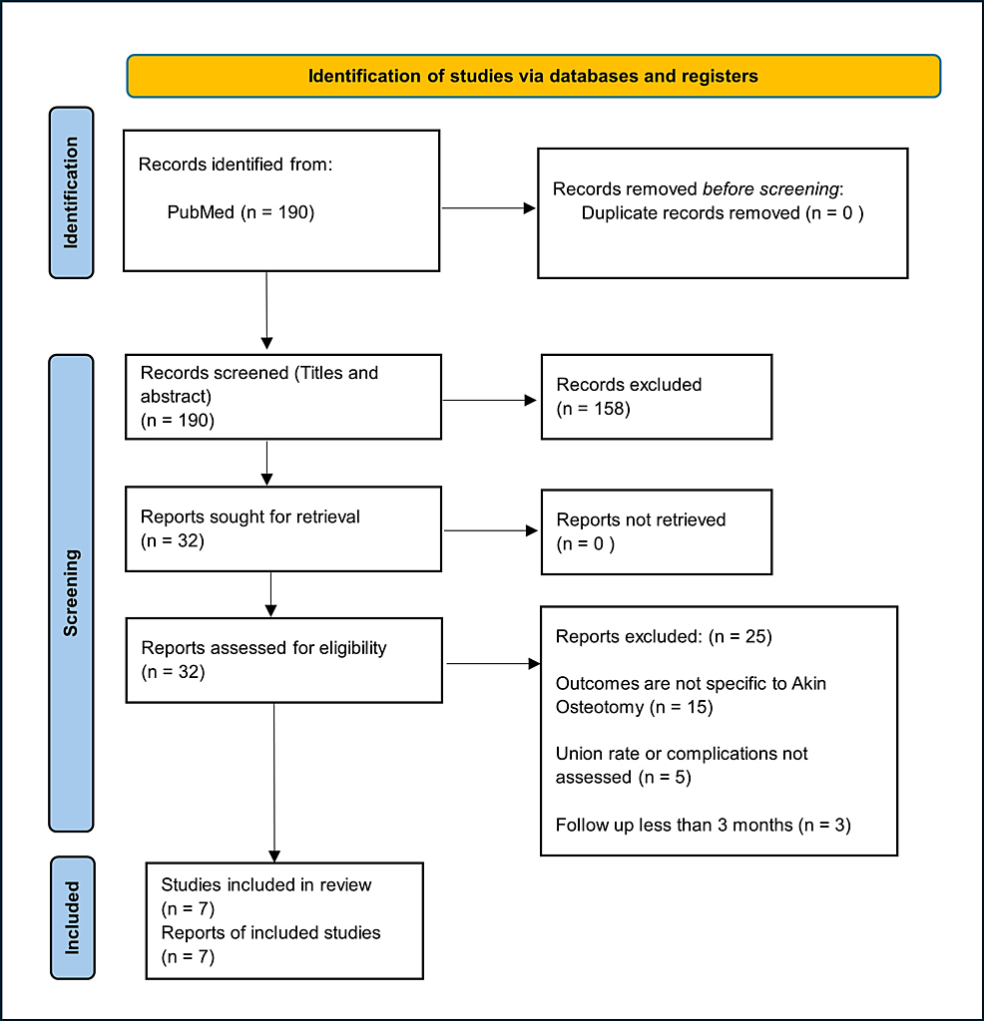
PRISMA flow diagram of the study selection process PRISMA: Preferred Reporting Items for Systematic Reviews and Meta-Analyses

We conducted a systematic literature search utilizing the PubMed database, employing the terms "Hallux Valgus," "Akin Osteotomy," "Screw," "Suture," and "Staples". Additionally, backward chaining was employed, delving into reference lists from located papers to augment the search. After screening titles and abstracts, 32 studies underwent review, and seven clinical studies were ultimately selected. Notably, all studies were retrospective, with three being comparative studies. Figure 1 illustrates the study selection process using the Preferred Reporting Items for Systematic Reviews and Meta-Analyses (PRISMA) flow diagram.

Inclusion Criteria

The inclusion criteria involved clinical studies involving screw, suture, or staple fixation techniques for the Akin osteotomy in patients aged 18 and above. Studies involving additional procedures had to address clinical outcomes and complications specifically related to Akin osteotomy; those lacking clear mention were excluded. Eligible studies were required to be conducted in English and published within the past 20 years.

We excluded older methods of Akin osteotomy fixation and percutaneous or minimally invasive techniques. Additionally, cadaveric and biomechanical studies were disregarded due to the absence of reported outcomes. We only considered studies that had a minimum follow-up duration of three months.

Data Synthesis

The following data were extracted and categorized based on the type of fixation (screw, staple, and suture): study design, year, intervention type, sample size, mean follow-up, bone union, mean union time, fixation-related complications, patient-reported outcome measures (PROMs), and radiographic angle measurements.

Results

The initial search yielded 190 articles. Among these, 158 were excluded during the abstract and title screening. Subsequently, 32 full-text articles underwent eligibility assessment, with 25 being rejected. Finally, seven studies met the criteria and were included in this review. The characteristics of each study are presented in Table 1.

**Table 1:** Characteristics of each study

Author	Study design	Type of fixation	Number of feet	Mean age (years) (range)	Sex (M/F)	Mean follow-up (months) (range)	Mean time to union (months) (range)	Bone union outcome (no. of feet)
Neumann et al. (2015) [15]	Retrospective	Staple	51	56.1 (19-82)	5/39	10.1 ± 3.9 (6.48-19.98)	2	100% (51)
Liszka et al. (2019) [16]	Retrospective Comparative	Screw	47	52 ± 34	5/42	12	2-4	100% (47)
		Staple	43	53 ± 35	3/40	12	2-4	100% (43)
		Suture	48	55 ± 30	3/45	12	2-4	100% (48)
Fazal et al. (2022) [18]	Retrospective Comparative	Screw	39	56	5/34	18.1 (6-32)	Unknown	100% (35)
		Staple	35	55.6	4/31	16.8 (6-30)	Unknown	100% (39)
Cullen et al. (2009) [19]	Retrospective	Suture	115	48 (25+67)	21/88	4 (3-12)	2	100% (115)
Matsumoto et al. (2022) [20]	Retrospective Comparative	Staple	39	69.9 ± 7.4	3/36	21.9 ± 13.3	2.70 ± 1.25	100% (39)
		Suture	58	64.9 ± 9.2	5/53	22.5 ± 13.6	2.55 ± 1.08	100% (58)
Tóth et al. (2010) [21]	Retrospective	Suture	22	49 (19-69)	1/17	26 (8-57)	1.5-2	100% (22)
Horner et al. (2023) [22]	Retrospective	Staple	93	51.5 (19-84)	12/78	14.2 (3.0-35.73)	Unknown	98.9% (92)

Seven retrospective studies, including three comparative studies, were eligible for analysis. The number of patients ranged from 22 to 109, with follow-up periods from 4 to 18.10 months. All studies showed a 100% union rate, except for Horner et al. [22], who reported a 98.9% union rate including both complete and partial union.

The duration for union varied across staple studies, ranging from 8 to 10.8 weeks), while in suture studies, it spanned from 6 to 10.20 weeks. However, it is important to note that not all reviewed studies specified the time to union, and there were variations in patient numbers. Additionally, there was no available time to union data for comparison in screw studies.

In the screw group, both Fazal et al. [18] and Liszka et al. [16] used one headless compression screw, with sizes ranging from 2.5 to 3 mm. In the staple group, one stable per case was utilized. Liszka et al. [16] and Matsumoto et al. [20] used a stainless-steel staple, while Neumann et al. [15] used EasyClip® Stryker memory nitinol staple. Similarly, Horner et al. [22] utilized SPEED® DePuy Synthes memory nitinol staple. In the suture group, an absorbable suture through a single tunnel was consistently utilized in all cases, except Matsumoto et al. [20], who used a non-absorbable suture (FiberWire, No.2). Notably, Liszka et al. [16] opted for a double tunnel technique in 28 out of 48 cases to augment stability.

Complications

The incidence of overall complications stood at 8.98%. An overview of the union rate and fixation-related complications of each technique type is presented in Table 2.

**Table 2:** Union rate and fixation-related complications of each technique

	Screws	Staple	Suture
No. of studies	2	5	4
No. of feet	86	261	243
Bone union (%)	100%	99.6%	100%
Fixation-related complications (feet)			
Metalwork irritation	3	3	-
Delayed union	-	-	-
Infection	-	1	1
Recurrence	-	1	1
Prominence	9	1	-
Reoperation	2	7	-
Lateral cortical breach	5	7	9
Local pain	-	3	-
Total complications	19	23	11

It is worth noting that metal prominence was higher in the screw group compared to the staple group. Fazal et al. [18] reported this complication in eight cases (20.51%) with screw utilization versus one case (2.86%) in the staple group, and this difference was statistically significant (p=0.02). Interestingly, Liszka et al. [16] reported only one case (1/47) of screw prominence in their study. Similarly, soft tissue irritation was reported in the screw and staple groups secondary to metalwork irritation and prominence, hence resulting in a higher reoperation rate when compared to the suture group.

Fazal et al. [18] reported reoperation in two (5.71%) cases in the staple group and one (2.56%) case in the screw group. Liszka et al. [16] demonstrated that one patient in the staple group experienced soft tissue irritation, while another in the screw group had discomfort due to a prominent screw. Both necessitated the removal of hardware six months after the initial procedure. In contrast, Horner et al. [22] reported that three patients (3.2%) needed to revisit the operating room due to trauma, infection, or pain.

Lateral cortical breech was reported in all fixation techniques. Matsumoto et al. [20] observed breakage of the lateral cortex in 10.3% of the cases, with seven out of 58 in the suture group and three out of 39 in the staple group; however, there was no significant difference between the two groups (p=0.74).

Among the patients in the seven studies, Horner et al. [22] documented only one case of deep wound infection necessitating surgery. Furthermore, Cullen et al. [19] noted that one patient with a superficial infection was successfully treated using oral antibiotics.

Radiological Outcomes

Due to the differences in the radiological parameters, conducting a comprehensive comparative analysis between the studies was unfeasible. Table 3 outlines the radiological measurements conducted in each study.

**Table 3:** Radiological parameters measured in each study HVIA: Hallux valgus interphalangeal angle, PDPAA: Proximal to distal phalangeal articular angle, DASA: Distal articular set angle

Authors	Type of fixation	Angle measured (mean)	Pre-op	Post- op
Neumann et al. [15]	Staple	HVIA	7.9°	-3.1°
Liszka et al. [16]	Screw	HVIA	12.1°	5.6°
	Staple	HVIA	11.7°	6°
	Suture	HVIA	12.5°	5.9°
Fazal et al. [18]	Screw	HVIA	13°	8.7°
	Staple	HVIA	12.9°	8.8°
Cullen et al. [19]	Suture	HVIA	8.2°	6.7°
Matsumoto et al. [20]	Staple	PDPAA	4.1°	-2.2°
	Suture	PDPAA	5.5°	-3.8°
Tóth et al. [21]	Suture	DASA	7.1°	-2.3°
Horner et al. [22]	Staple	Not recorded		

Fazal et al. [18] showed that the mean hallux valgus interphalangeal angle (HVIA) correction was 4.3° in the screw group and 4.1° in the staple group (p=0.73). Similarly, Liszka et al. [16] reported improvement in the HVIA across the three fixation groups with no statistically significant difference (p>0.05). Neumann et al. [15] reported improvement in the mean HVIA using staple fixation, demonstrating a substantial shift from 7.9° ± 3.4° before surgery to -3.1° ± 6.4° after surgery, showing statistical significance (p<0.001). On the other hand, Matsumoto et al. [20] compared the proximal to distal phalangeal articular angle (PDPAA) between the staple and the suture groups, showing significant improvement, but with no statistical significance between both groups (p=0.21). Additionally, Tóth et al. [21] used the distal articular set angle (DASA) showing a mean correction of 9.4° (SD 7.1, range 5-28) with his suture fixation technique.

PROMs

Because nearly all Akin osteotomies in the seven studies were coupled with additional procedures to correct hallux valgus, the PROMs documented in certain studies may not accurately represent the specific outcomes solely attributable to Akin osteotomies.

Liszka et al. [16] demonstrated that the mean score on the American Orthopaedic Foot and Ankle Society's (AOFAS) scale increased from 45 to 91 points in the staple group, from 42 to 90 points in the screw groups, and from 42 to 91 points in the suture group; however, there was no statistically significant difference between the three groups. Neumann et al. [15] analysed the visual analogue score (VAS) for the 44 patients who had staple fixation, and they found that the mean VAS dropped to (1.0 ± 1.2) from (4.4 ± 2.6.) with a statistically significant difference (p<0.001). He also reported that 78.7% of patients were completely satisfied, 14.9% were partially satisfied, and 6.4% were unsatisfied. Similarly, Horner et al. [22] reported the VAS scores for the 93 patients who underwent staple fixation showed considerably lower postoperative mean scores (1.3) compared to (2.1) preoperatively with a mean follow-up of 450 days. There was no statistically significant difference between the postoperative VAS scores of the healed Akin and partially healed cohorts (p=0.84).

Discussion

Hallux valgus is considered the most common forefoot deformity, with a global prevalence of 19% [1]. Surgical intervention is typically reserved for cases where conservative treatments have been ineffective. First metatarsal osteotomy has been widely accepted as the primary surgical approach for correcting hallux valgus deformity. However, the decision to include a proximal phalangeal osteotomy, such as the Akin osteotomy, remains a topic of debate. Thever et al. concluded that at a PDPAA > 8, patients receiving scarf and Akin osteotomy had a significantly better AOFAS score than patients with isolated scarf osteotomy at two years post-operatively [23]. Consistent findings favouring employing Akin osteotomy and metatarsal osteotomy are evident in the literature [4,24,25]. In their study of 54 cases of moderate to severe hallux valgus deformity, Park et al. observed a significant increase in HVIA after performing distal soft tissue release and proximal chevron metatarsal osteotomy. They concluded that, for optimal HV correction while preserving metatarsophalangeal joint (MTPJ) motion, an Akin osteotomy is frequently required to address associated hallux valgus interphalangeus (HVI) [25]. However, some studies reported uncertainties regarding the benefits of adding akin osteotomy [26].

Over the years, various techniques for fixing Akin osteotomy have been outlined and refined. The selection of a specific fixation method is influenced mainly by the surgeon's preference and the equipment. Unfortunately, a limited body of literature comprehensively reviews the outcomes of each fixation method. Despite Akin osteotomy typically exhibiting low complication rates and excellent bone union, our study compares the three most commonly employed methods today regarding complications, union rates, and cost, aiming to offer a more comprehensive understanding of the existing literature on these methods.

Screws have been utilized for fixing Akin osteotomy for a long time, providing robust fixation with a 100% union rate, as documented in our study. It is crucial to make an oblique osteotomy, as opposed to parallel to the joint line, to ensure that the screw is perpendicular to the osteotomy plane, thereby enhancing fixation [16]. Despite its advantages, the issue of screw prominence and soft tissue irritation remains a noted problem. In our analysis, nine out of 86 cases experienced prominent screws, leading to reoperation for screw removal in two cases [16,18]. Furthermore, there is a risk of lateral cortical breakage during screw insertion, with lateral cortical breach observed in five out of 86 cases fixed with screws [16,18]. It remains unclear whether these breaches occurred during screw insertion or the osteotomy procedures themselves.

Staple fixation has gained popularity recently due to its straightforward application and low complication rates. Various staples, from stainless steel to nitinol, have been developed over time. Notably, no significant clinical or radiological differences have been reported between the various types, including memory and heat compression staples [27]. Complications are not common but involve soft tissue irritation, which may require metal removal, which was noted in three out of 261 cases [6,18,22]. Additionally, damage to the articular cartilage could result from poor insertion technique. Lateral cortical breakage has been reported in a small percentage of cases, potentially associated with the force applied during staple hammering. To mitigate such occurrences, taking precautionary measures is crucial; these include drilling with a sufficient diameter, avoiding osteotomy close to the MTP joint, and ensuring the parallel insertion of the staple to the joint line [20].

Brahms introduced suture fixation for Akin osteotomy in 1981, utilizing absorbable sutures through the periosteum [28]. Subsequently, Melamed et al. outlined a single transosseous technique [29]. Zaragoza et al. employed a joint capsule suture fixation technique that eliminates the need for transosseous tunnels [30], while Liszka et al. [16] employed a double tunnel technique for cases requiring enhanced stability. Suture fixation has demonstrated positive outcomes, providing stable fixation while avoiding complications associated with metallic implants, such as irritation and the need for subsequent removal [13,19,21]. Despite the procedure's minimal instrumentation, some surgeons may find it technically demanding and require time to master. Caution is advised to prevent excessive opening of the gap at the osteotomy site, which could result in lateral cortical bridge fracture. In our analysis, lateral cortical breech occurred in nine out of 243 cases that underwent suture fixation. Matsumoto et al. reported 10 cases of lateral cortical breech, with seven in the suture group and three in the staple group. They observed that cases with lateral cortex breakage took significantly longer to achieve union than those without (13.3 +/- 6.4 vs. 10.1 +/- 4.3 weeks, p=0.04). However, there was no significant difference in the time to union between the suture and staple groups [20].

Considering the comparable outcomes across the three fixation groups, the cost may be a pivotal factor in selecting one technique. Fazal et al. illustrated this point by revealing that the total expense incurred for screws was £4,290, whereas for staples, it was £1,925 [18]. Liszka et al. conducted a thorough examination of implant prices and found that, in their hospital, suture fixation emerged as the most economical method. They reported an average cost of $75 for an 8 or 10 mm titanium headless screw, $3 for absorbable suture, and $80 for a metal alloy staple [16]. Similarly, Matsumoto et al. highlighted the cost-effectiveness of non-absorbable sutures compared to staples, with respective costs of $36 and $130 [20].

Our study has several limitations that merit consideration. Firstly, all the studies in our review were retrospective, introducing the potential for selection bias inherent in such designs without randomized controlled trials (RCTs). Additionally, the lack of a homogeneous population across the seven studies, particularly regarding medical comorbidities and chronic diseases, such as diabetes mellitus and rheumatoid arthritis, may impact the precision of results regarding outcomes and complications. Moreover, because Akin osteotomy is usually combined with other surgical operations, it makes it difficult to attribute outcomes solely to Akin osteotomy. Lastly, the indications for performing Akin osteotomy were often unclear, possibly due to the retrospective nature of the studies, with many cases involving its inclusion as part of bunion correction procedures.

## Conclusions

This systematic review presents a singular attempt to compare outcomes in Akin osteotomy employing the most recent fixation methods. Our findings suggest that there are no significant differences in terms of bone union or complications across the three fixation groups. Nevertheless, cost-effectiveness considerations may lean towards favouring the use of the suture technique.

While our study did not specifically examine the indications for Akin osteotomy, we recognize the importance of well-designed RCTs investigating this topic, which remains a debate among foot and ankle surgeons.
